# Comprehensive Analysis of a Ferroptosis Pattern and Associated Prognostic Signature in Acute Myeloid Leukemia

**DOI:** 10.3389/fphar.2022.866325

**Published:** 2022-05-17

**Authors:** Zelong Cui, Yue Fu, Zongcheng Yang, Zhenxing Gao, Huimin Feng, Minran Zhou, Lu Zhang, Chunyan Chen

**Affiliations:** ^1^ Department of Hematology, Qilu Hospital, Cheeloo College of Medicine, Shandong University, Jinan, China; ^2^ Center of Stomatology, The First Affiliated Hospital of USTC, Division of Life Sciences and Medicine, University of Science and Technology of China, Hefei, China

**Keywords:** leukemia, ferroptosis, prognosis, immune status, drug sensitivity

## Abstract

Ferroptosis is a widespread form of programmed cell death. The environment of cancer cells makes them vulnerable to ferroptosis, including AML cells, yet the specific association between ferroptosis and AML outcome is little known. In this study, we utilized ferroptosis-related genes to distinguish two subtypes in TCGA cohort, which were subsequently validated in independent AML cohorts. The subtypes were linked with tumor-related immunological abnormalities, mutation landscape and pathway dysregulation, and clinical outcome. Further, we developed a 13-gene prognostic model for AML from DEG analysis in the two subtypes. A risk score was calculated for each patient, and then the overall group was stratified into high- and low-risk groups; the higher risk score correlated with short survival. The model was validated in both independent AML cohorts and pan-cancer cohorts, which demonstrated robustness and extended the usage of the model. A nomogram was constructed that integrated risk score, FLT3-ITD, TP53, and RUNX1 mutations, and age. This model had the additional value of discriminating the sensitivity of several chemotherapeutic drugs and ferroptosis inducers in the two risk groups, which increased the translational value of this model as a potential tool in clinical management. Through integrated analysis of ferroptosis pattern and its related model, our work shed new light on the relationship between ferroptosis and AML, which may facilitate clinical application and therapeutics.

## Introduction

Programmed cell death (PCD), a process regulated by both extracellular and intracellular mechanisms, is indispensable for natural development and homeostasis of organisms (Bedoui et al., 2020). Ferroptosis, which was first proposed as a nonapoptotic form of programmed cell death in 2012 (Dixon et al., 2012), is now regarded as one of the most prevalent and conventional forms of cell death (Jiang et al., 2021). Functionally, ferroptosis is characterized by iron dependency and excessive reactive oxygen species (ROS) with lipid peroxidation. Morphologically, ferroptotic cells exhibit smaller than normal mitochondria, fewer mitochondria crista, and ruptured outer mitochondrial membrane. These features distinguish ferroptosis from other forms of PCDs including necrosis, apoptosis, and autophagy (Xie et al., 2016). Moreover, ferroptosis has a profound impact on a multitude of diseases, ranging from neurodegenerative disorders to ischemia–reperfusion injury in organ transplantation and cancer (Stockwell et al., 2020).

Iron, a trace element in the human body, is necessary for maintenance of cancer cells as well as normal cells. Increased cellular iron import and reduced iron export is common in many cancers. This iron addiction phenomenon has been found in solid tumors such as neuroblastoma (Floros et al., 2021), ovarian (Basuli et al., 2017), and prostate cancers (Tesfay et al., 2015). Elevated cellular iron levels promote the proliferation of cancer cells but also make them more vulnerable to a ferroptosis inducer, due to its stimulation to ROS production (Ma et al., 2016; Floros et al., 2021). On the contrary, GPX4, the key regulator of ferroptosis, uses glutathione (GSH) as an antioxidant to neutralize harmful ROS to defend cells from ferroptosis (Yang et al., 2014).

Acute myeloid leukemia (AML) is the most common type of acute leukemia in adults. The incidence is 4.3 per 100,000 in America. Most patients diagnosed with *de novo* AML can reach remission after induction chemotherapy. However, except the specific genetic background such as PML/RARa fusion gene, the long-term survival of AML patients remains unsatisfying.

The malignant hematopoietic cells of AML show similar iron accumulation and dependency (Liu et al., 2014; Benadiba et al., 2017; Wang et al., 2019), like their solid tumor counterparts. Moreover, AML patients often have an elevated serum ferritin level (Lebon et al., 2015), not only a sign of excess iron in the body, but it also correlates with a worse disease prognosis. This iron overload status also helps with immune evasion in AML (Aurelius et al., 2012). These factors indicate that AML has significant potential susceptibility to ferroptosis. Several therapies targeting ferroptosis or combining with ferroptosis to sensitize chemotherapy in AML have achieved some progress. For example, DHA and APR-246 were reported to induce ferroptosis in the AML cells (Du et al., 2019; Birsen et al., 2021). The ferroptosis inducer erastin can enhance the anticancer activity of cytarabine and doxorubicin in AML (Yu et al., 2015). Intriguingly, recent research highlighted ironomycin, a ferroptosis inducer, to disrupt the mitochondrial metabolism and overcome venetoclax resistance in AML (Garciaz et al., 2022). However, the relationship between ferroptosis-related genes and clinical outcome in AML remains elusive.

In this study, we collected AML samples from seven independent cohorts for analysis. We identified two distinct subtypes in TCGA cohorts and validated this distribution in external GEO cohorts. The two subtypes, with prognostic significance, were inconsistent in baseline information, immune cell infiltration, and mutation burden. From these subtypes, we built a risk score system which robustly predicted patients’ survival. We also found that the score is associated with various clinical characteristics, anticancer immune status, and chemotherapy response. These results highlight the role of ferroptosis in AML and contribute to the further investigations of molecular mechanisms and medical interventions.

## Materials and Methods

### Data Acquisition

Data for patients with AML were retrieved from online databases, including five GEO databases (GSE10358, GSE14468, GSE37642, GSE71014, and GSE106291, *n* = 1432), TCGA-LAML (*n* = 150), and BeatAML (*n* = 197). The samples were sifted when they are in replicate, or their survival time is zero. A total of 13,322 genes were retrieved from these datasets, and their expression values were obtained for further operation. For platform-independent purpose, we performed a rank-based transformation on microarray and RNA-Seq data. As previously described (Warnat-Herresthal et al., 2020), gene expression values were transformed from microarray intensities or RNA-Seq counts to their respective ranks in a gene-wise way, meaning all gene expression values per gene were given a rank by their order from the lowest value to the highest. The five GEO databases were merged into a single large database for this transformation, while TCGA and BeatAML were transformed as two independent datasets. Ferroptosis-associated genes were retrieved from FerrDb (http://www.zhounan.org/ferrdb/). A protein–protein interaction (PPI) network was constructed by the information from STRING.

### Consensus Clustering and Robustness Verification

Based on the expression data of these genes in TCGA and merged GEO cohorts, we performed unsupervised clustering analysis using the “ConsensusClusterPlus” package in R. The K-means method was used to identify the number of clusters, and the analysis included 1000 iterations to ensure the stability of the classification (Wilkerson and Hayes, 2010). The “Nbclust” package was used to find the robust cluster number and “PCA3D” package to depict the three-dimensional distribution of the principal component analysis (PCA) score of these samples (Charrad et al., 2014; Shao R. et al., 2021). The IGP score was calculated in the “ClusterRepro” package to measure the reproducibility of gene expression clusters in TCGA and merged GEO datasets (Wu et al., 2021).

### Differentially Expressed Gene Analysis

To identify genes associated with each cluster, the differentially expressed genes (DEGs) between two ferroptosis-related clusters in TCGA cohort were determined using the limma package in R. The volcano plot of DEGs was drawn using the ggplot package, and the heatmap of DEGs was drawn using the pheatmap package.

### Gene Set Variation Analysis

We performed GSVA enrichment analysis by the gsva packages (Hänzelmann et al., 2013). We applied “c2. cp.kegg. v7.2. symbols” and “h.all.v7.2. symbols” from MSigDB to complete GSVA analysis. Adjusted *p* < 0⋅05 was considered statistically significant between different clusters by the limma package.

### Immune Cell Infiltration

We used the CIBERSORTx algorithm and single sample gene set enrichment analysis (ssGSEA) to quantify the proportions of immune cells in AML samples (Liang et al., 2020; Wang et al., 2021a). For CIBERSORTx, normalized gene expression data were uploaded to the web portal with LM22 signature and 1,000 permutations. For ssGSEA (single sample GSEA), the gene expression of AML samples was used as an input in the gsva package to generate scores of 29-type immune cells for these samples.

### Construction and Validation of Risk Model

The survival package was used to perform univariate Cox regression analysis of differentially expressed genes between clusters 1 and 2. The “Glmnet” package was used for least absolute shrinkage and selection operator (LASSO) analysis. The risk score is calculated as follows:

$$\overset{k}{\underset{i=1}{\sum }}\beta iXi$$

where k, βi,and Xi represent the number of signature genes, the coefficient index, and the gene expression level, respectively. The cut-off value used to determine risk groups in this article was −0.197 as a median risk score of the TCGA cohort.

### Drug Sensitivity Prediction

Drug sensitivity data along with the gene expression data of AML cell lines were obtained from the Cancer Therapeutics Response Portal (CTRP v2.0, https://portals.broadinstitute.org/ctrp).The risk scores were calculated to separate risk groups. The lower area under the curve (AUC) of the dose–response curve indicated increased drug sensitivity (Yang et al., 2021). Then, we applied the “pRRophetic” R package to compute the AUC of several common chemotherapeutic drugs and some ferroptosis inducers in the TCGA cohort (Geeleher et al., 2014). These drugs include cytarabine, doxorubicin, azacytidine, decitabine, erastin, RSL3, ML210, vorinostat, venetoclax, and esmodegib.

### Construction of the Nomogram

We used the “rms” R package to build the nomogram and the calibration chart. The calibration chart was used to evaluate the performance of the nomogram. Decision curve analysis (DCA) was used to evaluate the clinical implementation of the nomogram by quantifying the net benefits at different threshold probabilities using the “dcurves” package.

### Patients and Sample Preparation

Bone marrow samples were acquired from patients with newly diagnosed AML (*n* = 10) and iron-deficiency anemia (*n* = 10) who were treated at the Department of Hematology, Qilu Hospital of Shandong University in Jinan, China. The mononuclear cells were isolated from the samples and stored at −80°C.

### RNA Extraction and Real-Time Quantitative PCR

The total RNA was extracted by Trizol reagent (Invitrogen, Carlsbad, CA, United States) according to the manufacturer’s protocol. Subsequently, the extracted RNA was reversely transcribed using the PrimeScript RT reagent Kit with gDNA Eraser (Takara, Japan). The cDNAs were subjected to SYBR Green-based real-time PCR analysis. The primers used in real-time PCR assays are listed in Supplementary Table S5.

### Statistical Analysis and Cut-Off Value

The correlation coefficients per two genes in Figure 2A were computed by Spearman’s and distance correlation analysis. Log-rank tests were utilized to identify the significance of differences in survival curves. The time-dependent ROC curves and the area under curves (AUC) were derived using the timeROC packages. Comparisons of the integrated area under the curves (IAUC) in these ROCs were implemented with the iauc.comp package, and *p*-value was derived from the Mann–Whitney test. The RCircos package was used to plot the copy number variation landscape of 13 related genes in 23 pairs of chromosomes. For continuous variables, Student’s t-test and Wilcox test were performed between two groups. One-way ANOVA and Kruskal–Wallis test were used to compare three or more groups. For categorical variables, Chi-square tests were used. The universal cutoff score between two risk groups is −0.197 from TCGA median risk score. All statistical *p*-values are two-sided, and *p* < 0.05 represents statistical significance. Asterisk signal: * *p* < 0.05; **p < 0.01; ***p < 0.001.

## Results

### Identification of a Robust Classification Pattern in Two Acute Myeloid Leukemia Cohorts With 47 Ferroptosis-Related Genes

**FIGURE 1:**
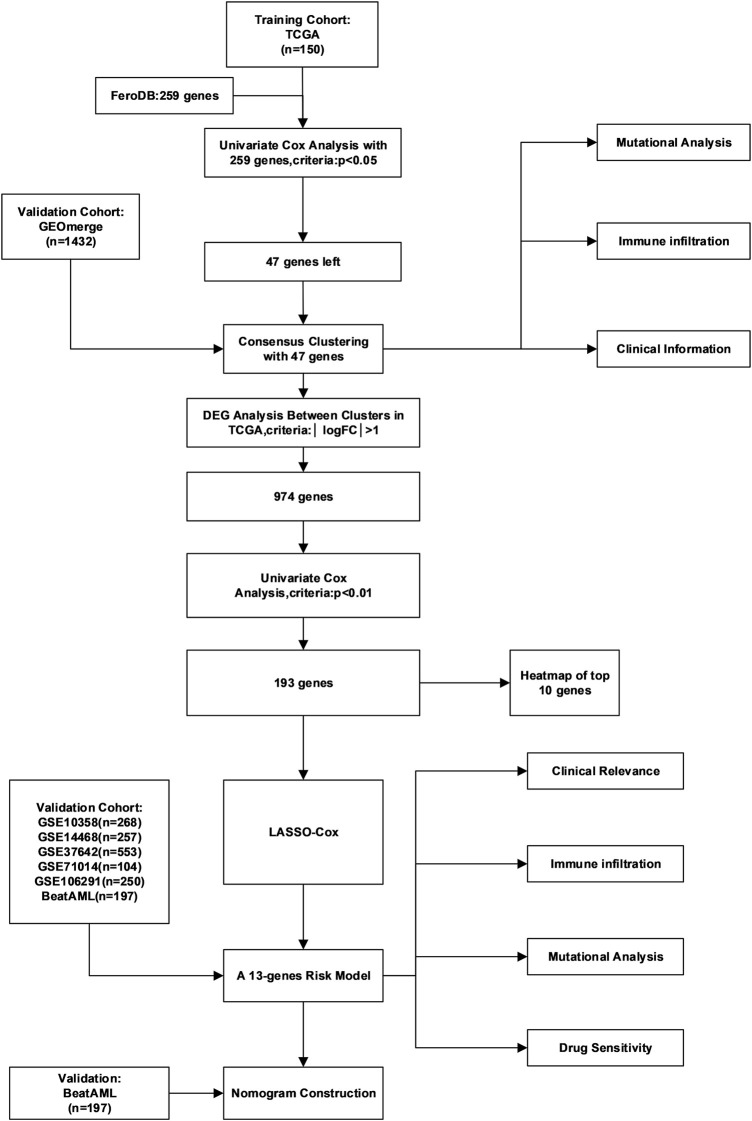
Detailed flow chart of the whole research.

**FIGURE 2:**
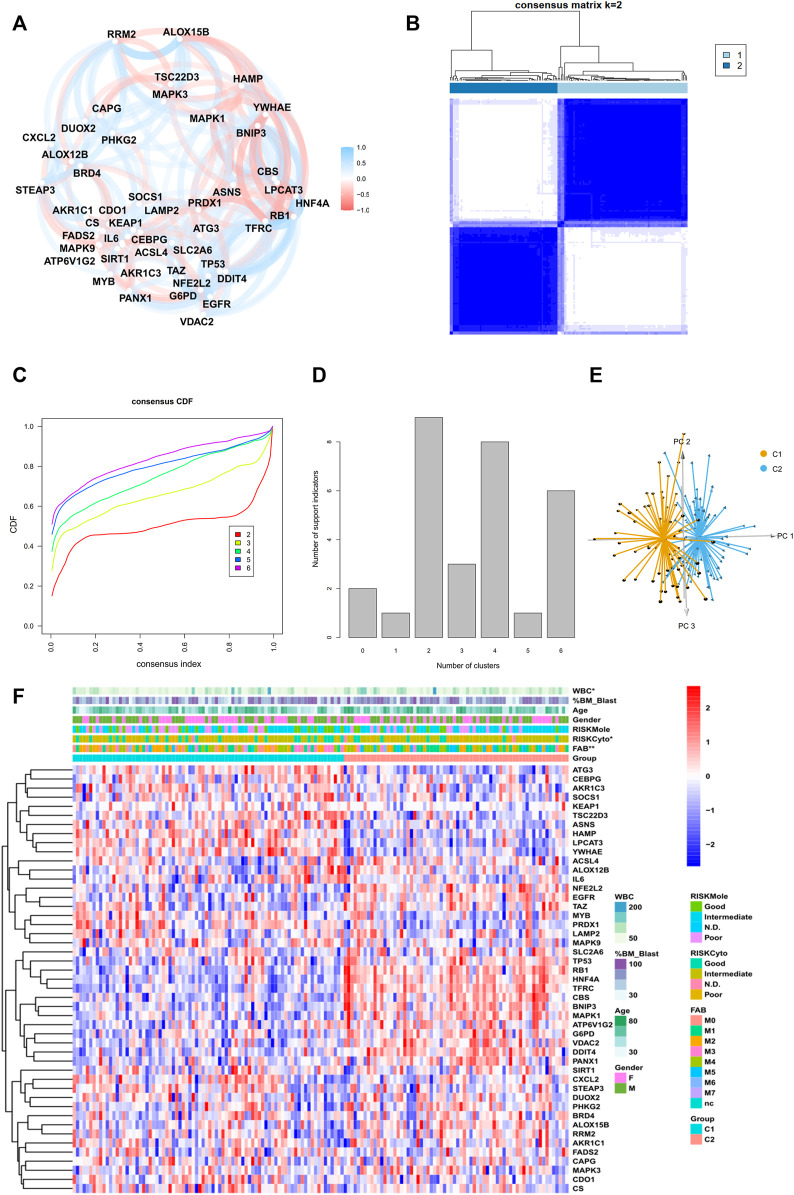
Identification of two subtypes in the TCGA cohort. **(A)** Correlation plot of 47 genes, **(B)** consensus matrix of two subtypes, **(C)** CDF curve, and **(D)** indication barplot of k-value used for consensus clustering.**(E)** PCA plot of two subtypes.**(F)** Heatmap of 47-gene expression with clinical information in the TCGA cohort. (Categorical variables, Chi-square tests; continuous variable, Wilcoxon tests *p < 0.05; **p < 0.01; ***p < 0.001).

The general workflow is depicted in Figure 1A. To identify ferroptosis-related genes that have prognostic implications, we performed a univariate Cox regression on 259 ferroptosis-related genes in the TCGA dataset. A list of 47 genes with prognostic significance were selected to conduct further analysis (Supplementary Figure S1A). The transcriptional expression correlation and PPI of these genes are depicted to reveal the inner relationship between these genes (Figure 2A, Supplementary Figure S1B). The unsupervised clustering divided the TCGA dataset patients into two subtypes based on gene expression data (Figure 2B). This division was supported by the consensus CDF curve and optimal number in Nbclust (Figures 2C,D). The PCA analysis was conducted by PCA3D to validate the distinct divergence of these two clusters (Figure 2E). This clustering pattern was further validated in the merged GEO dataset, which also showed a double-subtype division (Supplementary Figures S2A–D). The similarities of C1 and C2 subtypes in reciprocal datasets were validated with in-group proportion (IGP) algorithm. The IGP score of C1 was 88.0% and that of C2 was 89.3% in the TCGA dataset and 83.5 and 80.0% in the GEO-merge cohort, respectively (all *p*-values<0.001).

### Differences in Immune Cell Infiltration, Clinical Characteristics, and Pathway Enrichment Between Two Ferroptosis-Related Subtypes

**FIGURE 3:**
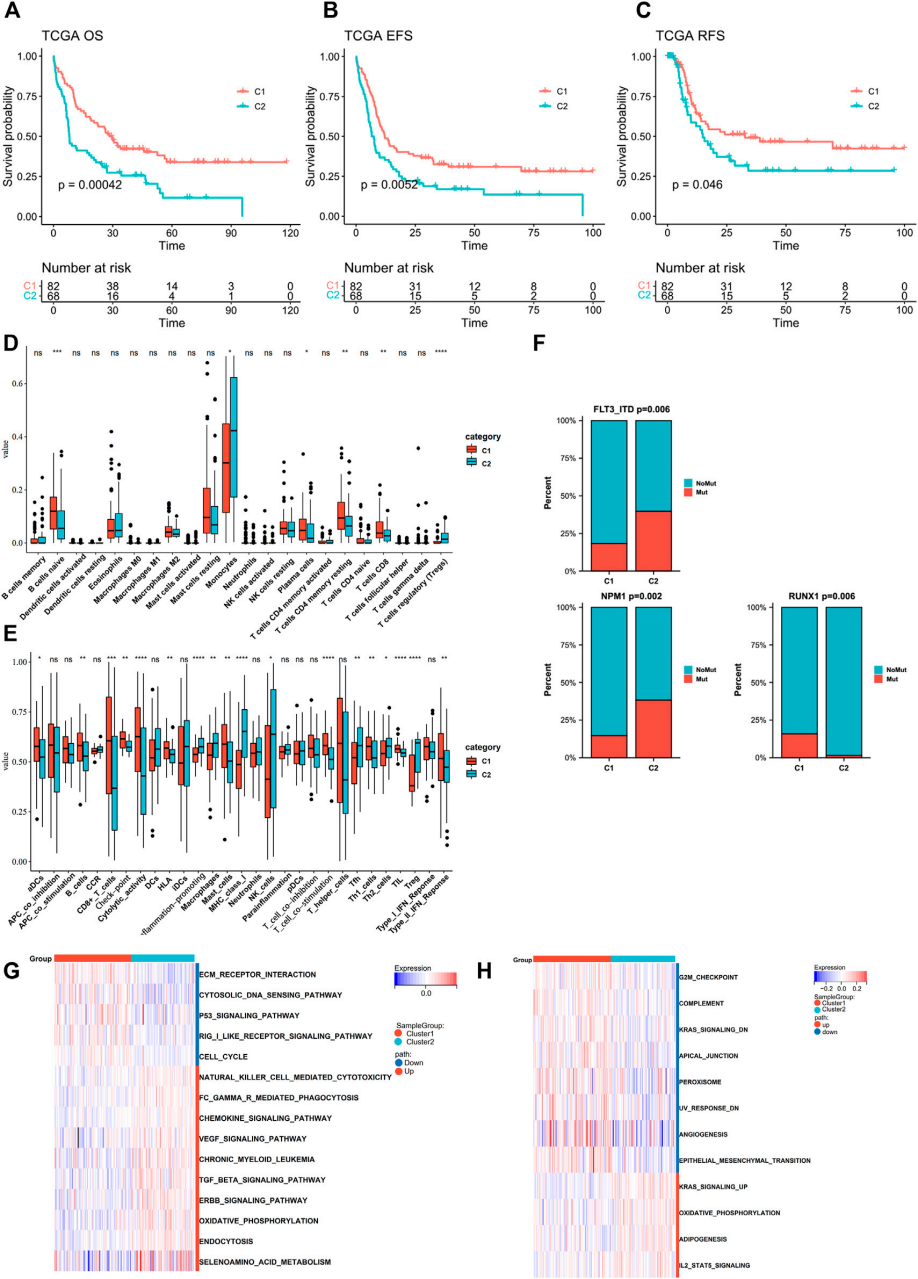
Assessment of differences in prognosis, immune elements, and pathway enrichment between the two subtypes in TCGA cohort. **(A**–**C)** Kaplan–Meier survival analysis of the two clusters in TCGA (OS, overall survival; EFS, event-free survival; RFS, relapse-free survival. C1 *n* = 82, C2 *n* = 68; log-rank p-test). **(D**,**E)** Comparison of the difference in immune cell fraction and immune response between the two subtypes, with CibersortX and ssGSEA algorithm (Wilcoxon tests). **(F)** FLT3-ITD, NPM1, and RUNX1 mutation distribution between the two subtypes (Chi-Square tests). **(G**,**H)** Heatmaps of GSVA-based KEGG and Hallmark analysis of the two subtypes (Wilcoxon tests).

The clinical parameters were also compared between two subtypes (Figure 2F, Supplementary Table S1). Cluster C2 consisted of more FAB M5 patients and fewer M3 patients and an elevated level of WBC, while Cluster C1 had more samples in the cytogenetically “favorable” risk group. The survival analysis of the TCGA dataset and GEO-merged dataset showed that C2 demonstrated an inferior prognosis (Figures 3A–C; Supplementary Figure S3E). Immune escape is a major issue in AML, leading to treatment resistance and relapse. To elucidate the immune response in each subtype, we conducted tumor infiltration analysis. The results revealed that compared with cluster C2, cluster C1 had high percentages of CD8^+^ T cells, tumor-infiltrated lymphocytes, and a stronger Type I IFN response, and a low percentage of Treg cells using CibersortX and ssGSEA algorithms (Figures 3D,E, Supplementary Figure S3F,G). These results were confirmed in both the TCGA and GEO-merged datasets.

Recurrent gene mutation in AML, such as *FLT3-ITD* and *RUNX1*, can contribute to pathogenesis and disease progression, thus influencing the outcomes (Marcucci et al., 2011; Heath et al., 2017; Daver et al., 2019). Therefore, we analyzed gene mutation status in TCGA samples (Supplementary Figure S1C). Point mutations were extracted from the TCGA dataset, of which the 10 highest (*FLT3-ITD, NPM1, DNMT3A, IDH2, IDH1, CEBPA, RUNX1, TET2, TP53*, and *WT1*) were compared between the two groups. C2 showed higher frequencies of *FLT3-ITD* and *NPM1* mutations (C1vsC2: *FLT3-ITD*:19–37%; *NPM1*:15–39%), whereas the *RUNX1* mutation was more frequent in C1(C1 vs. C2: *RUNX1*:15–1%) (Figure 3F, Supplementary Figure S2H).

To investigate the underlying mechanisms that regulated biological function and clinical and immunological characteristics between two clusters, we performed GSVA analysis of Hallmark and KEGG in the TCGA dataset (Figures 3G,I). The C2 signaling pathways such as ERBB, TGF-beta, and KRAS were upregulated. In contrast, the cancer-inhibiting pathways such as TP53 and G2M checkpoints were downregulated. Additionally, C2 showed upregulation in selenoamino acid metabolism and downregulation in peroxisome. Both of them can regulate ferroptosis, in addition to TP53 pathway. GSEA analysis also found ERBB and TGF-beta activation in the C2 cohort (Supplementary Figure S3A).

### Development and Validation of a 13-Gene Signature Derived From Ferroptosis-Related Subtypes

To further decipher the cause for the differences between the two patterns, we conducted a differentially expressed gene (DEG) analysis and found a distinct expression variation of the two subtypes in TCGA datasets. (The cutoff was |log2FoldChange| >1 and FDR<0.05.) Of 974 genes, 806 were upregulated, and 168 were downregulated (Supplementary Table S7). The GO and KEGG analyses depicted genes that were enriched in a vast number of cancer pathways ranging from solid tumors to hematological malignancies (Supplementary Figures S3B–C). Furthermore, the analysis also revealed an enrichment in the lipid metabolic process, which was reported to regulate ferroptosis throughout initiation and progression of cancer (Li and Li, 2020).

**FIGURE 4:**
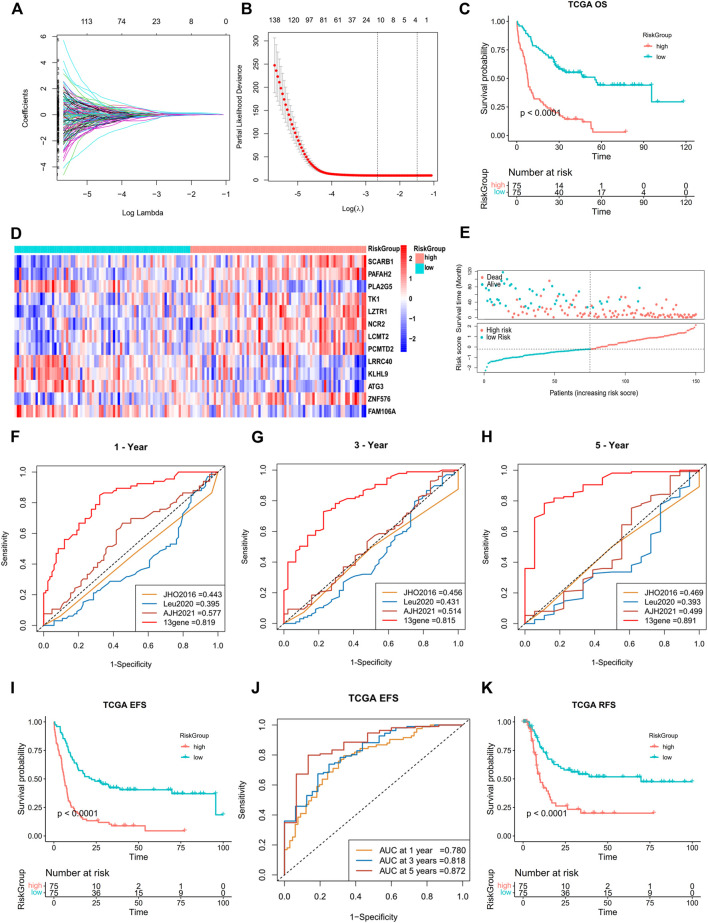
Construction of a 13-gene prognostic signature by DEGs in two subtypes. **(A**,**B)** Least absolute shrinkage and selection operator (LASSO) regression analysis with ten-fold cross validation to determine the lambda number. **(C)** OS analysis of the risk group in TCGA dataset (log-rank p-test). **(D)** Heatmap of 13-gene expression in different risk groups (Wilcoxon tests). **(E)** Dot plot showing the distribution of risk score in patients with different survival time and status in TCGA. **(F**–**H)** Prognostic ROC AUC comparison in the 13-gene signatures with other known signatures in the training datasets (TCGA) for 1, 3, and 5 years. **(I**,**J)** EFS analysis and ROC of the risk group in TCGA dataset (log-rank *p*-test). **(K)** OS analysis of the risk group in the GSE10358 dataset (log-rank *p*-test).

**FIGURE 5:**
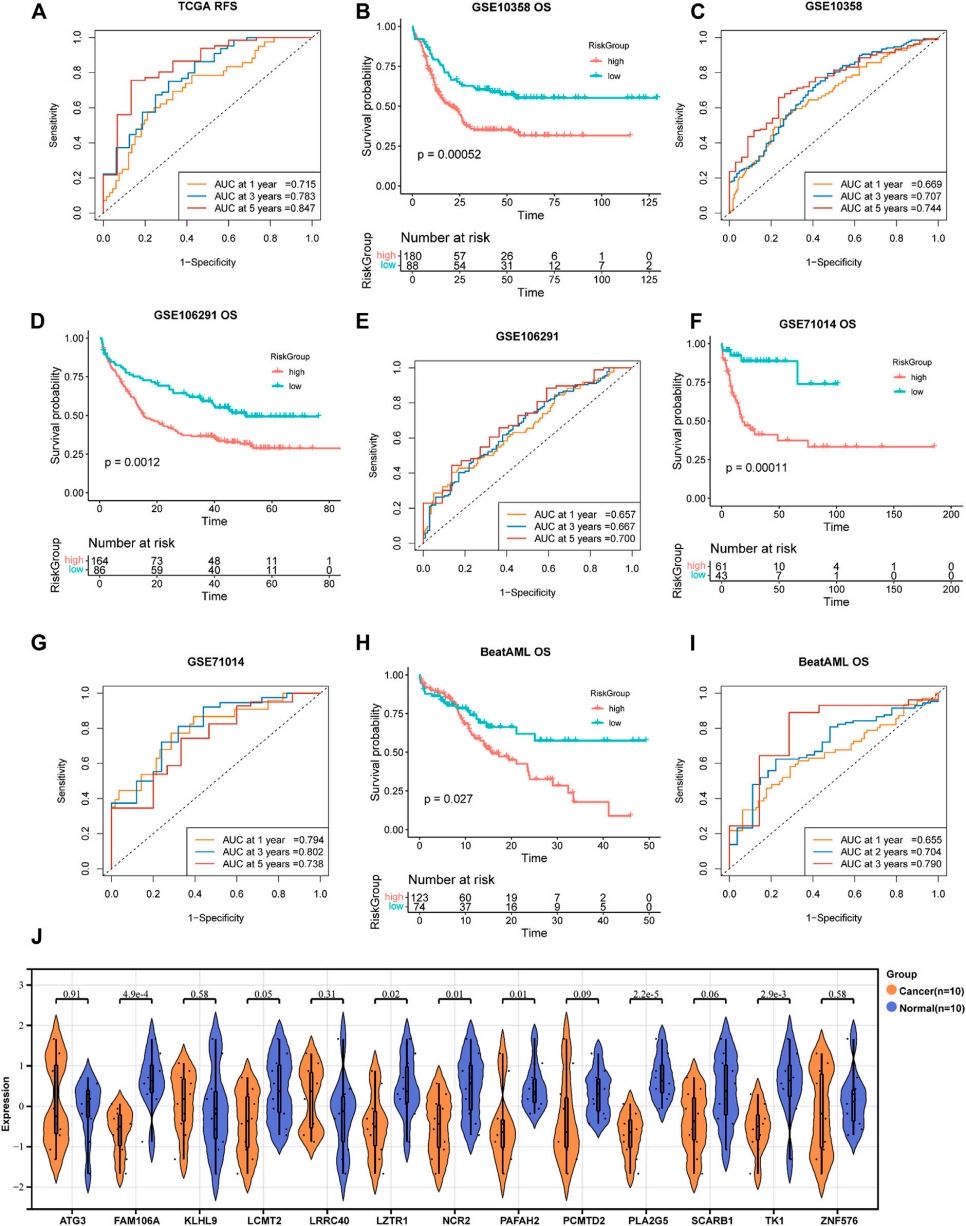
Validation of the 13-gene prognostic signature in different datasets. **(A**,**B)** RFS analysis and ROC of the risk group in TCGA dataset. Reciprocal survival analysis and ROC of GSE71014 **(C**,**D)**, GSE106291 **(E**,**F)**, and BeatAML **(G**,**H)** datasets were illustrated. 5I ROC of GSE10358 survival analysis displayed in Figure 4K. **(J)** RT-PCR-detected RNA expression of 13 genes in our own samples, 10 AML *de novo*, and 10 IDA as normal control samples.

A total of 193 DEGs meeting specific criteria (univariate cox analysis *p* < 0.01) were screened out (Supplementary Figure S3D, Supplementary Table S2). Then, these genes were analyzed using a LASSO Cox algorithm to construct a prognostic model (Figures 4A,B). In this procedure, the TCGA dataset was established as the training cohort, while the other GEO cohorts and BeatAML cohort were used as the validation cohort. Eventually, 13 genes were identified, and the risk score was calculated according to the given coefficients: Risk score = ATG3 * (−0.28134) + FAM106A * (−0.10016) + KLHL9 * (−0.07429) + LCMT2 * (−0.4437) + LRRC40 * 0.1902 + LZTR1 * 0.2291 + NCR2 * 0.20767 + PAFAH2 * 0.32442 + PCMTD2 * 0.14762 + PLA2G5 * (−0.44088) + SCARB1 * 0.39573 + TK1 * (−0.51048) + ZNF576 * 0.20382 (Supplementary Table S3). The risk score and the risk group defined by its median value in the TCGA datasets had a statistically superior capability to predict the overall survival (OS) compared to other previously reported prognostic signatures [AJH 2021 (Chen et al., 2021), JCO 2013 (Marcucci et al., 2014), and JHO 2016 (Wilop et al., 2016)] (iAUC-13gene: 0.828, iAUC-AJH2021: 0.526, iAUC-Leu2020: 0.405, and iAUC-JHO2016: 0.492; all the *p*-values in comparison of iAUCs between 13-gene and three reciprocal known signatures were <0.001), (Figures 4C,E–H). Moreover, the risk score was excellent in predicting event-free survival (EFS) and relapse-free survival (RFS) in the TCGA dataset (Figures 4I,J, Figures 5A,B). These results were confirmed in the validation analysis using the GEO datasets (GSE10358, GSE71014, and GSE106291) and BeatAML datasets, which were estimated by reciprocal ROCs and Kaplan–Meier plots (Figure 4K, Figures 5C–I). The profile of the 13 genes were characterized, including their expression levels between clusters, their prognostic significance in the K-M plots, and their mutation status in TCGA datasets (Figure 4D, Supplementary Figures S4A–M, S1D). The dependency of the 13 genes composing the score in the AML cells was also analyzed using the DepMap portal (https://depmap.org/portal/) (Supplementary Figures S5A,B). We also utilized our own AML *de novo* clinical samples with IDA samples as normal control to detect the expression of 13 genes (Figure 5J).

**FIGURE 6:**
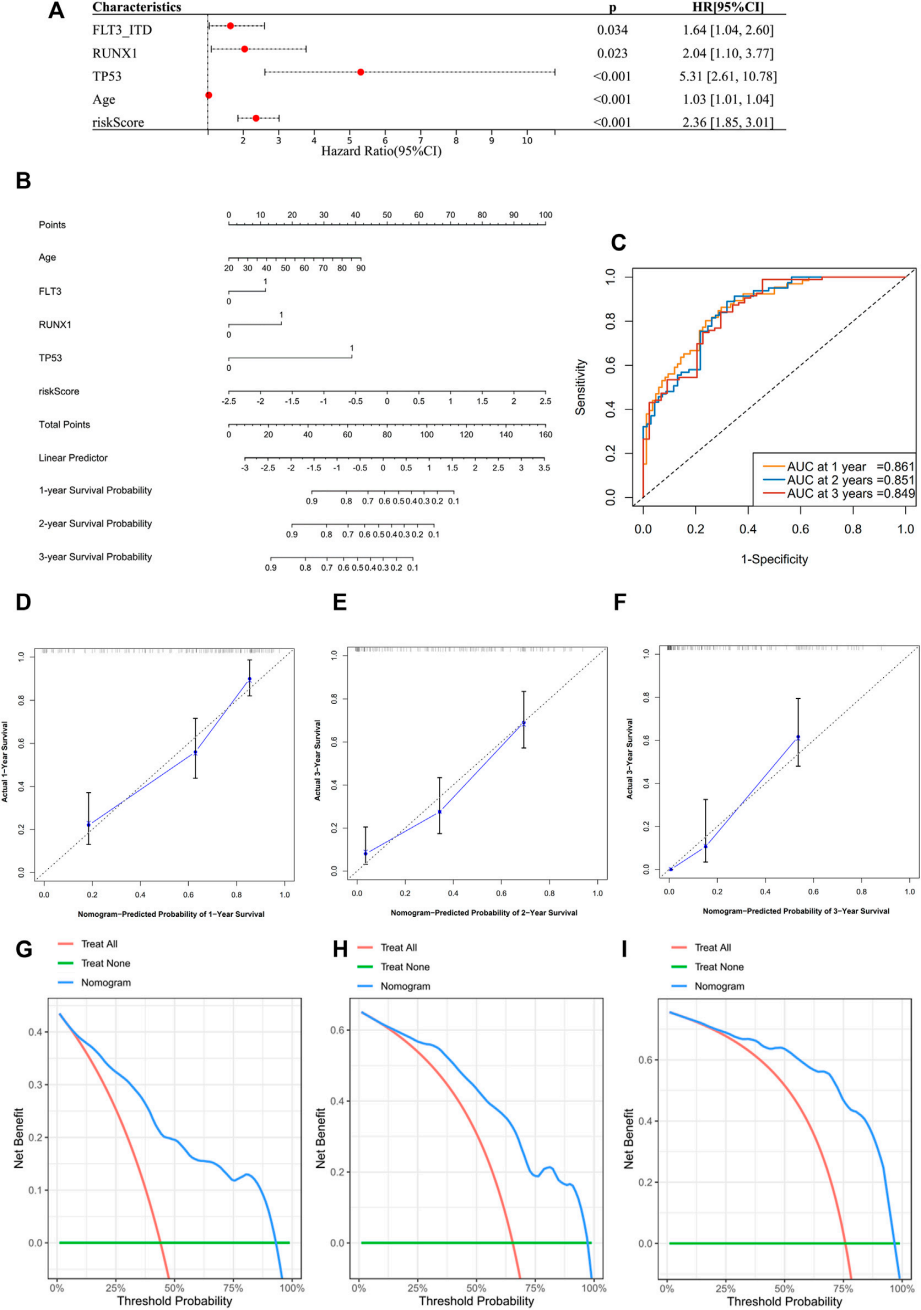
Construction of a prognostic nomogram in TCGA datasets. **(A)** Multivariate analysis to validate independent prognostic factors from the previous multivariate Cox screening in Supplementary Figure S4N. **(B)** Nomogram for clinical diagnosis based on age, mutation status, and risk score. **(C)** ROCs for the nomogram. **(D**–**F)** Calibration plots for predicting survival at 1, 2, and 3 years. The x-axis represents the predicted survival probability from the nomogram, and the y-axis represents the actual survival probability. **(G**–**I)** Decision curve analysis of the nomogram for 1-, 2-, and 3-year risk. The x-axis represents the threshold probability, and the y-axis represents the net benefit. The green line represents the assumption that no patients died at 1, 2, or 3 years. The red line represents the assumption that all patients died at 1, 2, or 3 years, and the blue line represents the prediction model of the nomogram.

The next step was to further enhance the risk score to facilitate its clinical application. We performed a multivariate Cox regression analysis incorporating the following variables: the 10 most frequent mutations, gender, and age in the TCGA dataset. In addition to risk score and age, mutations of FLT3-ITD, TP53, and RUNX1 were proved to be independent impact factors for the OS (Supplementary Figure S4N). These five variables were then subjected to a new multivariate Cox regression to develop a nomogram (Figures 6A,B). The AUC of ROC curves showed accuracy of this nomogram at 1-, 2-, and 3-year time points, with the AUCs of 0.861, 0.851, and 0.849, respectively (Figure 6C). Segmental lines at different times for this nomogram were close to 45-degree angles, indicating a good prediction performance (Figures 6D–F). The decision curves at 1, 2, and 3 years were also analyzed. Within the threshold of the probability range (1-year: 4–92%; 2-year: 7–97%; and 3-year: 9–96%; Figures 6G–I), the nomogram showed the net benefit compared to an all or none strategy. Furthermore, the nomogram was validated using the BeatAML cohort (Supplementary Figures S6A–H), which showed similar prediction potential. More recently reported ferroptosis-related gene signatures were also compared to our 13-gene score, but ours was statistically superior (iAUC-13gene: 0.824, iAUC-JCMM: 0.437, and iAUC-BJBMS: 0.646, *p*-values between iAUC of 13-gene and two new signatures were <0.001) (Huang et al., 2021; Shao W. et al., 2021) (Supplementary Figures S7A–C).

To systematically analyze the prognostic significance of the 13-gene risk score in pan-cancer, we conducted a univariate Cox analysis in TCGA data of 33 types of malignancies, including LAML (Supplementary Figure S7D). A higher risk score was identified as a negative prognostic biomarker for the five independent TCGA cohorts. Apart from LAML, one hematological (DLBC, diffuse large B-cell lymphoma) and three other tumor cohorts (cervical squamous cell carcinoma and endocervical adenocarcinoma (CESC), skin cutaneous melanoma (SKCM), and thyroid carcinoma (THCA)) were enrolled. Therefore, the risk score showed moderate scalability in other types of cancer.

### Higher Risk Score Indicated Worse Clinical Characteristics and Lower Immune Cell Infiltration

**FIGURE 7:**
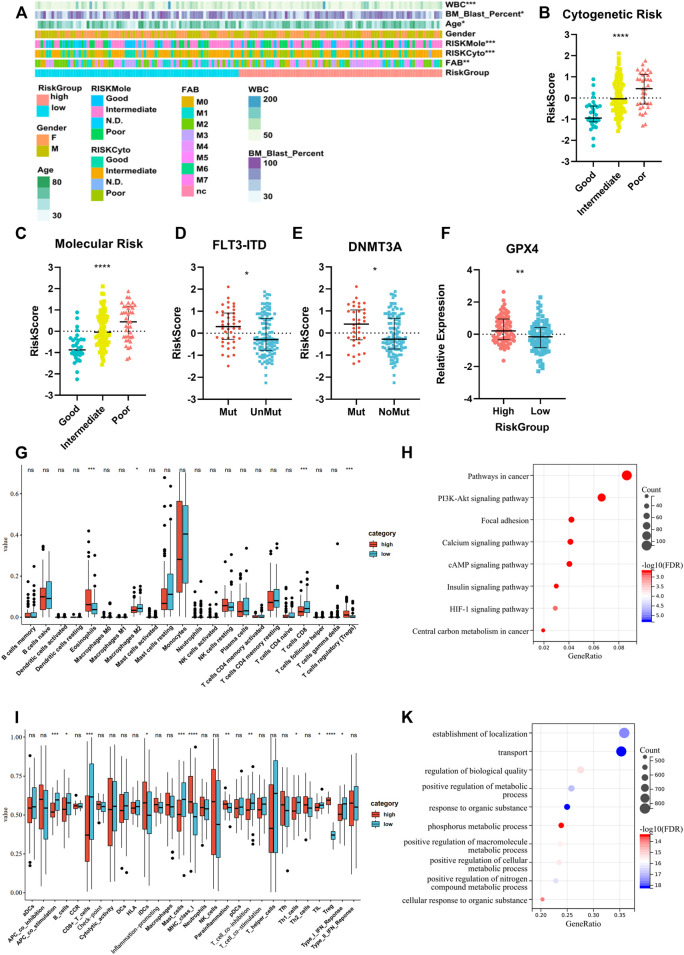
Analysis of differences in clinical characteristics, immune cell proportion, and functional enrichment of patients in different risk groups. **(A)** Clinicopathological features were compared between low-risk and high-risk groups (Categorical variables, Chi-square tests; continuous variable, Wilcoxon tests *p < 0.05; **p < 0.01; ***p < 0.001). **(B**,**C)** Comparison of the risk scores of patients with different cytogenetic and molecular risk types (Kruskal–Wallis test). **(D**,**E)** Comparison of the risk scores between patients with or without FLT3-ITD and DNMT3A mutations (Wilcoxon test). **(F)** Comparison of GPX4 expression between patients in high- and low-risk groups (Student’s t-test). **(G**,**I)** Different immune cell fraction and immune response between the two subtypes, with CibersortX and ssGSEA algorithm (Wilcoxon test). **(H**,**K)** GO and KEGG pathway analysis for differentially expression genes between the two risk groups (Fisher’s exact-test).

We compared the clinical characteristics of patients in different risk groups in the TCGA dataset. The heatmap shows that the distributions of age, percentage of bone marrow blast cells, peripheral WBC counts, FAB type, and cytogenetic and molecular risk classifications in the high- and low-risk groups were significantly different (Figure 7A, Supplementary Table S4). We found that the risk score was significantly altered among the samples of different cytogenetic and molecular risk (Figures 7B,C), with a higher risk score appearing in the high-risk group. Patients harboring *FLT3-ITD* and *DNMT3A* mutations had a higher risk score than patients without these mutations (Figures 7D,E). We also compared the GPX4 expression level between different risk groups and found higher expression in the high-risk group (Figure 7F). Differences of immune signatures in the high- and low-risk groups were also compared, and we found that CD8^+^ T cells, costimulation T cells, TILs, and type-I IFN response were expressed at higher levels in the low-risk group and Tregs at higher levels in the high-risk group (Figures 7G,I). The comparison of KEGG and GO analysis between the two risk groups determined that DEGs were enriched in cancer pathways: PI3K-Akt pathway, HIF-1 pathway, and central carbon metabolism in cancer. Also notably, several metabolic processes were enriched as well (Figures 7H,K).

### Two Risk Groups Showed Inconsistent Sensitivities With Different Drugs

**FIGURE 8:**
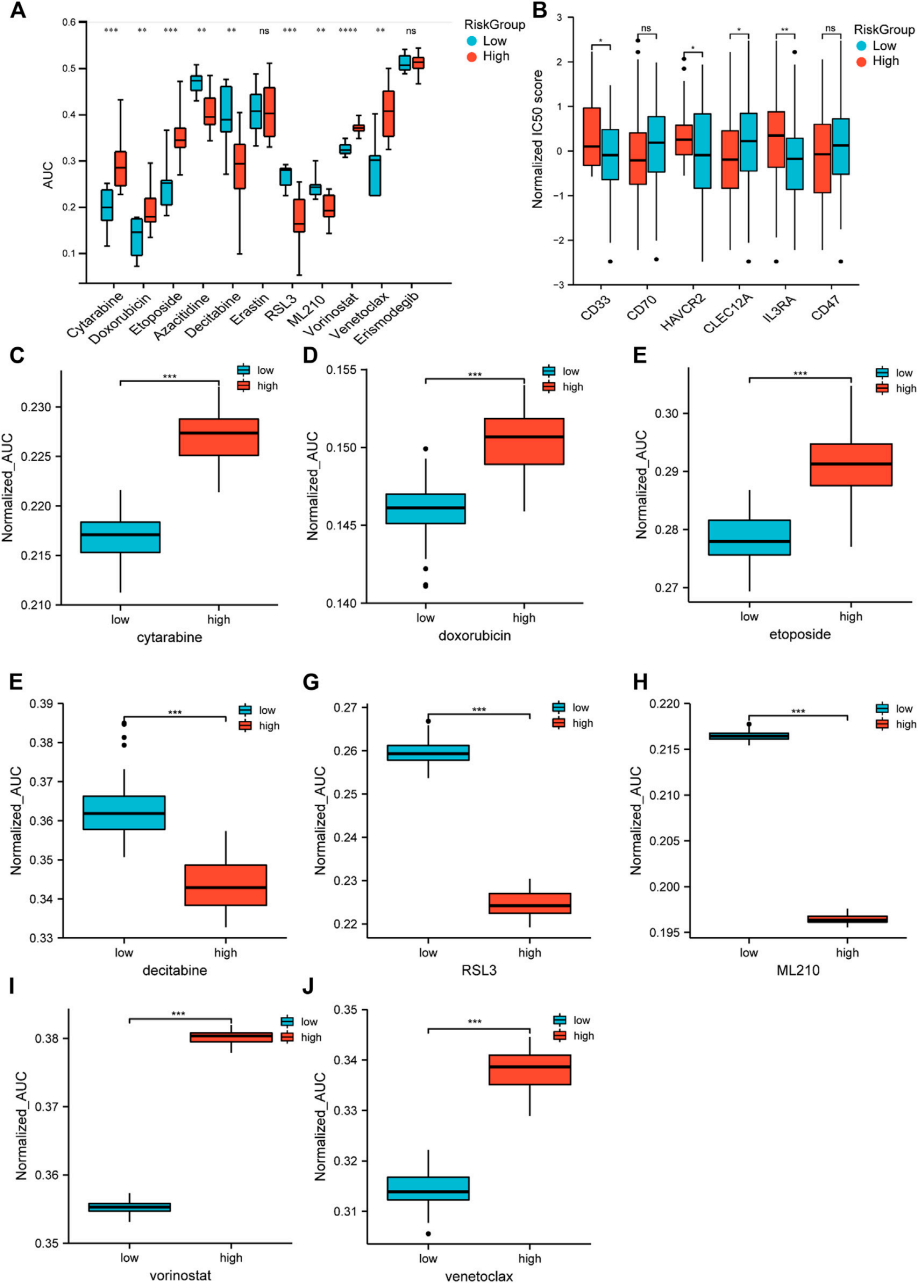
Drug response prediction in cell lines and samples. **(A)** Cell-based drug response of different risk groups. Risk scores were calculated by 13-gene expression in CTRP’s AML cell lines. Then, the score divided them into two risk groups based on the universal cut-off. Lower AUC value means better drug response. **(B)** CD33, CD70, HAVCR2, CLEC12A, IL3RA, and CD47 expression in different risk groups (Student’s t-tests). **(C**–**J)** Estimated drug response in different risk groups (Wilcoxon tests). Panel 8A data came from cell lines. Panel 8B–J data were obtained from TCGA datasets.

Chemotherapy is the main treatment for AML patients, although drug resistance is a major obstacle. Therefore, we applied CTRP (Cancer Therapeutics Response Portal) data to correlate drug resistance and sensitivity with the risk groups. The low-risk group was more sensitive to the traditional cytotoxic drugs, such as cytarabine, doxorubicin, and etoposide, and the BCL-2 inhibitor venetoclax. The high-risk group showed less resistance to hypomethylating agents (HMA), such as decitabine and azacytidine, and HDAC (histone deacetylase) inhibitors, such as vorinostat. Moreover, we found that the sensitivity of RSL3 and ML210, which were known to inhibit GPX4 and thereby inhibit ferroptosis, were significantly correlated with the high-risk group (Figure 8A). Then, we utilized an algorithm called pRRophetic to propagate predictions from CTRP data into TCGA samples. The patient sensitivity and resistance data were correlated with their counterparts in the cell lines (Figures 8C–J). These results indicated that the response to chemotherapeutic medications differed according to the two risk groups. Although ICI (immune checkpoint inhibitor) failed to provide cures in patients with AML (Bewersdorf and Zeidan, 2020), targeting specific antigens expressed in AML has been intensively investigated and gained some progress (Dohner et al., 2021). Therefore, we further analyzed the expression of AML-specific immunotherapy targets in the two risk groups (Figure 8B). The protein-coding genes CD33, HAVCR2, and IL3RA showed higher expression in the high-risk group. These findings distinguished unique chemotherapy reactivity and immune antigen expression in the two groups.

## Discussion

AML is an aggressive hematopoietic disease caused by rapid clonal expansion of undifferentiated malignant cells in bone marrow. Despite novel regimens and risk-stratified therapeutics, the 5-year survival of AML patients at diagnosis was still less than 30% in the years ranging from 2011 to 2017, according to the NIH SEER database (https://seer.cancer.gov/statfacts/html/amyl.html). There is still an urgent need to improve the clinical outcome. The iron overload nature of AML makes induction of ferroptosis an enticing treatment option. Intriguingly, the ferroptotic pattern in AML and its underlying impact remains largely unknown.

Precision medicine has brought revolutionized changes in cancer treatment in the recent years. By integrating multidimensional data from biological and clinical sources, heterogeneous cancer samples can be more accurately divided into subtypes for individualized treatment. Increasing evidence established the subgroups of patients based on their molecular profiles, representing distinct phenotypes, prognosis, and therapy responses. For instance, Zhou et al. (2019) classified the colorectal cancer patients into high- and low-risk groups according to an autophagy-related gene pattern, and intensive intervention is required for the high-risk group patients. Based on the RNA N6-methyladenosine-related regulator expression, the gastric cancer patients can be divided into three subtypes with distinct immune phenotype and prognosis. Patients from high-risk subtypes were more resistant to immunotherapy (Zhang et al., 2020). Devin et al. (2022) developed a signature of key effectors of iron metabolism based on the gene expression profile and identified that a subgroup of patients with DLBCL (diffuse large B-cell lymphoma) with poor outcome could benefit from an iron-targeted therapy. The present study identified two robustly distinct ferroptosis-related patterns, Cluster 1 (C1) and Cluster 2 (C2). These two cohorts had distinct differences not only in prognosis but also in baseline clinical status, immune response, immune cell fraction, and pathway activation. Compared with C1, C2 had a low level of CD8^+^ T cells and costimulating T cells and higher levels of regulating T cells (Treg), which are the signs of immune evasion. The type-II IFN response, an anticancer signature, is also relatively weak in C2. Evidence has shown that Treg cells can protect themselves from ferroptosis and maintain immune suppression. The pathway enrichment analysis also revealed that C2 had an upregulated selenoamino acid metabolism and downregulated the P53 pathway. GPX4, a selenoprotein produced from selenoamino acid, assists ferroptosis resistance (Yang et al., 2014), whereas inhibition of the P53 pathway can lead to tumorigenesis (Chu et al., 2019). Additionally, upregulated procancer pathways (ERBB, TGF-beta, VEGF, and KRAS) and procancer mutations are seen more frequently in C2. Aggregating all of these findings, we proposed two patterns, of which C2 had more passive immune response, more resistance to ferroptosis, and more proliferation potency; these culminated a significant reduction in the overall, event-free, and relapse-free survival.

Derived from differentially expressed genes in two clusters, we identified 13 genes (*ATG3, FAM106A, KLHL9, LCMT2, LRRC40, LZTR1, NCR2, PAFAH2, PCMTD2, PLA2G5, SCARB1, TK1*, and *ZNF576*) which, through univariate and LASSO Cox analysis, were used to construct a prognostic model. Some of these genes have previously been reported to participate in ferroptosis through various pathways, including *ATG3* (Zhou et al., 2020), *SCARB1* (Foulks et al., 2008; Kono et al., 2008), PAFAH2 (Foulks et al., 2008; Kono et al., 2008), and *PLA2G5* (Samuchiwal and Balestrieri, 2019; Tang et al., 2021). *KLHL9* (Lee et al., 2015), *LCMT2* (Wang et al., 2018), and *LZTR1* (Abe et al., 2020) have been reported to influence cancer progression in different contexts. *TK1* has long been used as a diagnostic and prognostic marker in AML (O'Neill et al., 2007), and *PCMTD2* (Atkins et al., 2019) and *LRRC40* (Zeybek et al., 2019) have been used as diagnostic or prognostic markers in other types of cancer. *NCR2* reportedly indicated a low-function state of NK cells in AML (Fauriat et al., 2007; Venton et al., 2016). To date, it appears that little is known about the function of *FAM106A* and *ZNF576* in biological processes; further experimental work will be required.

The 13-gene prognostic model described previously was robust, as demonstrated by several independent validation cohorts and comparisons with other reported gene signatures. Moreover, the prognostic value appeared not only to be applicable to AML but several other cancer types as well. The analysis also confirmed that several mutations were associated with the prognosis in AML. Multivariate Cox regression identified *FLT3-ITD, RUNX1,* and *TP53* mutations as independent prognostic factors, and a nomogram comprising risk score, these mutations, and patient age was developed, thereby translating this biological research to a clinical application.

AML is a heterogenous disease, in which data-based identification of patients for personalized therapy should provide a positive impact on the clinical outcome (Burd et al., 2020). As a screening database, CTRP portal provides multi-omic information including transcriptome data and drug response information in the cancer cell lines. With the help of pRRophetic algorithm, these *in vitro* screening data can be put into *in vivo* drug sensitivity prediction (Wang et al., 2021b). In our study, the risk groups derived from the risk score also showed diversities in cellular and organismic sensitivity to chemotherapeutic agents and ferroptosis inducers; the low-risk group was more sensitive to common cytotoxic agents (cytarabine and doxorubicin), whereas the high-risk group was more sensitive to HMA agents. The high-risk group showed a higher *GPX4* expression and was more sensitive to ferroptosis inducers directly targeting GPX4, RSL3, and ML210. This was reasonably expected considering the iron addiction nature and high ROS level in AML cells (Wang et al., 2019; Wei et al., 2020). Unlike other solid tumors and lymphoma, immune checkpoint inhibitors have gained moderate efficacy in the treatment of AML. In addition, several surface epitopes have proved to be potential targets of AML. Therefore, we explored the expression of several AML-specific immune targets (CD33, CD70, HAVCR2 (TIM-3), CLEC12A, IL3RA (CD123), and CD47) between different groups. CD33, HAVCR2, and IL3RA were expressed higher in the high-risk group, whereas CLEC12A was expressed higher in the low-risk group. These indicated divergent targets in different risk groups. Therefore, this risk score can provide therapeutic indication and support its translational value.

In this study, we identified and validated two ferroptosis subtypes in AML. These subtypes exhibited distinct heterogeneity in baseline information, immune response, functional pathways, and clinical outcomes. Furthermore, a 13-gene scoring system was constructed that demonstrated a reliable prognostic efficacy with an indication of chemotherapy sensitivity and anticancer immunity. We believe that these findings will increase the knowledge of ferroptosis and have the potential to facilitate more precise therapeutic intervention in AML.

## Data Availability

The datasets presented in this study can be found in online repositories. The names of the repository/repositories and accession number(s) can be found in the article/Supplementary Material.
