# Supplementary figures and images for: Comprehensive Analysis of a Ferroptosis Pattern and Associated Prognostic Signature in Acute Myeloid Leukemia
Source: Front Pharmacol. 2022 May 17;13:866325. doi: 10.3389/fphar.2022.866325 (PMC9152364; doi:10.3389/fphar.2022.866325)

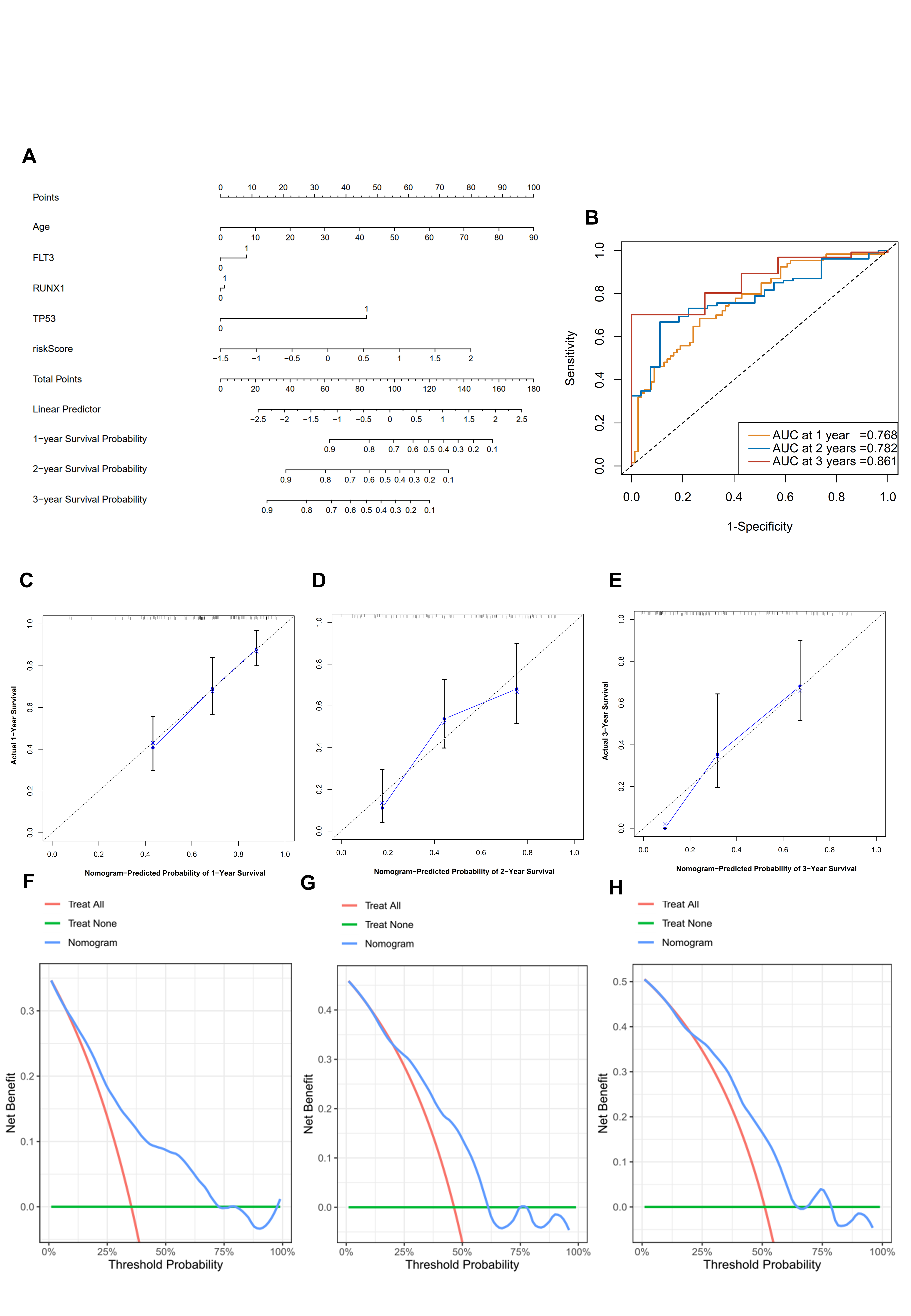

Supplement: Supplementary file 3 [file Image6.TIF]

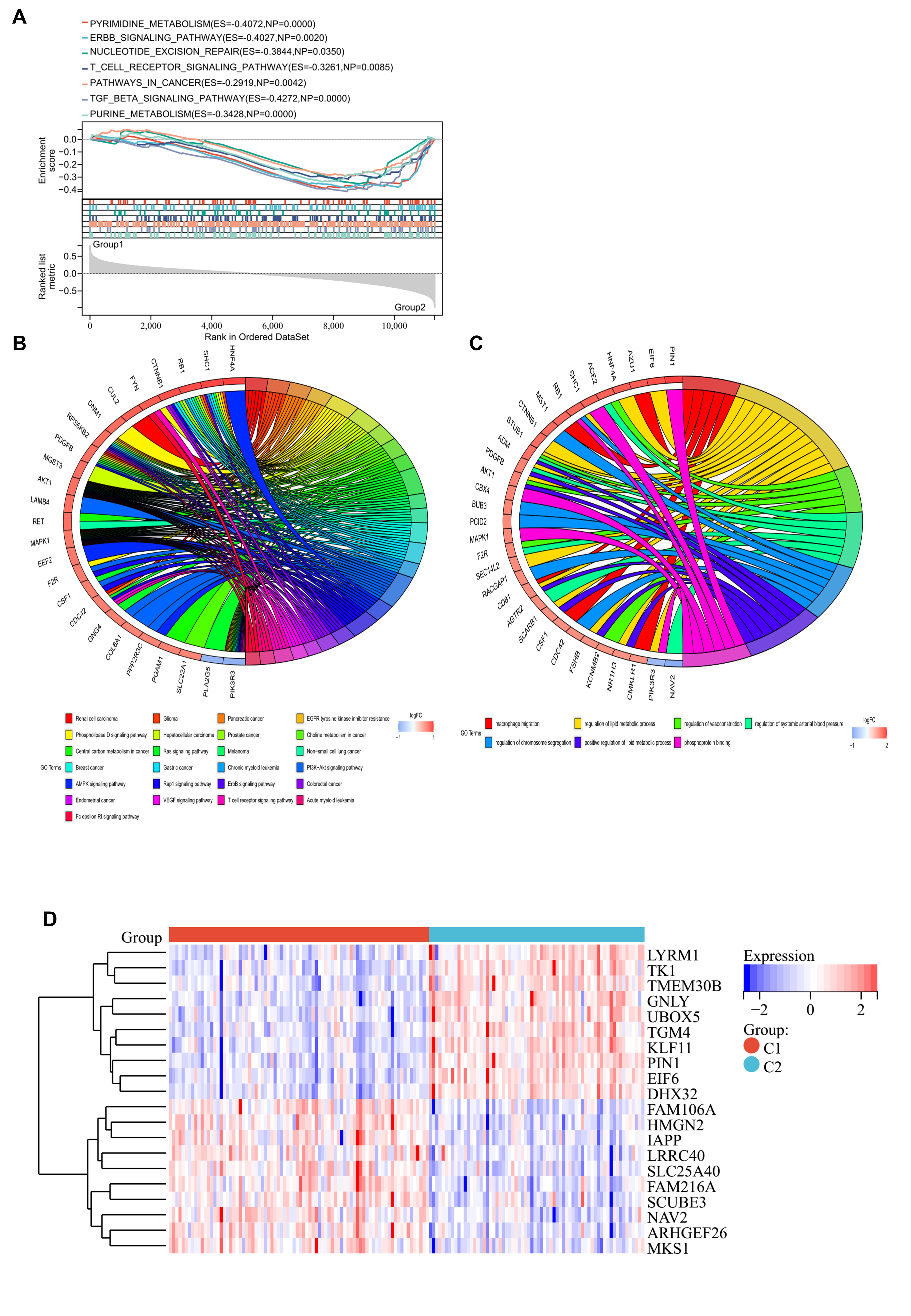

Supplement: Supplementary file 4 [file Image3.TIF]

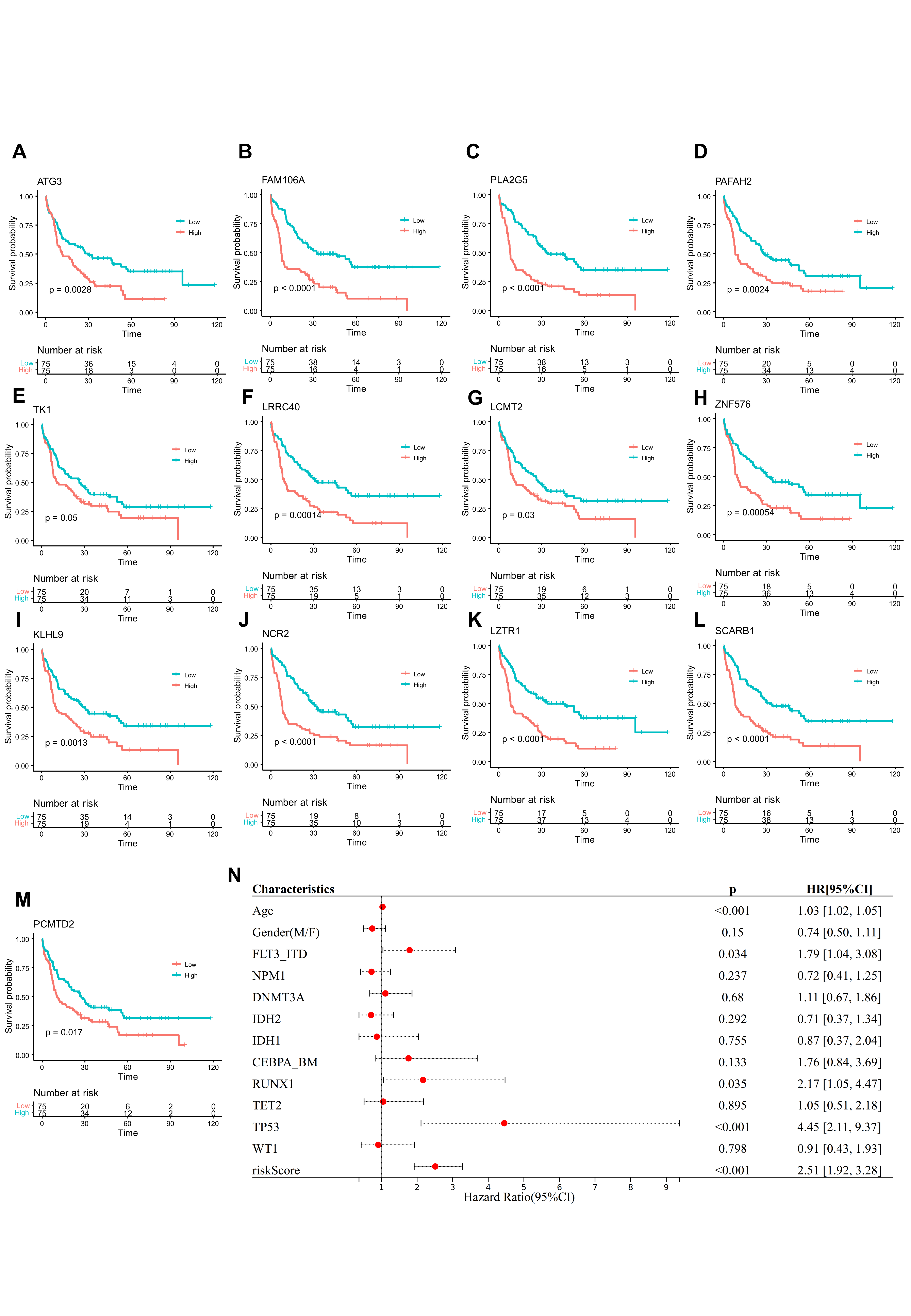

Supplement: Supplementary file 5 [file Image4.TIF]

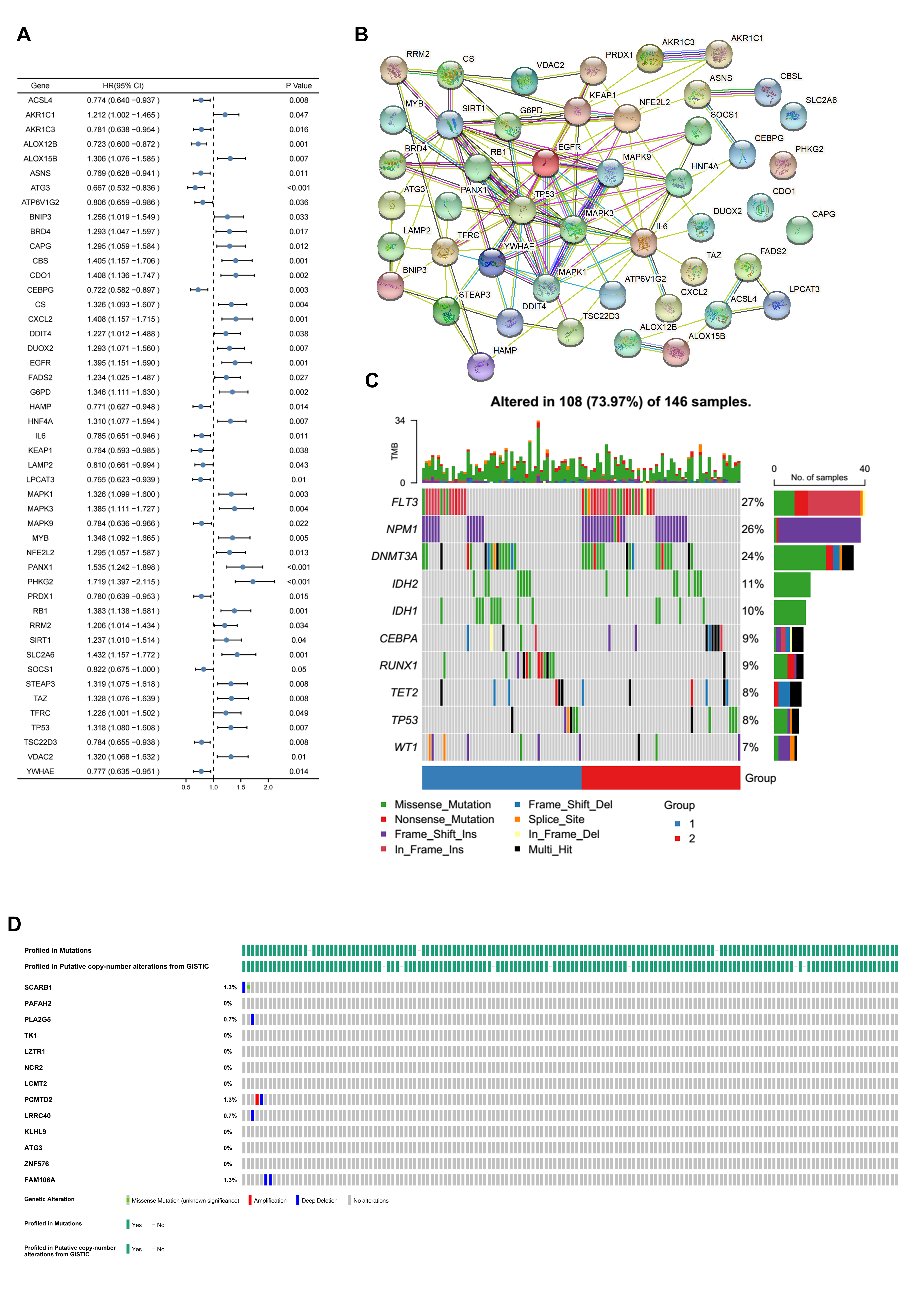

Supplement: Supplementary file 6 [file DataSheet1.zip › FigS1.TIF]

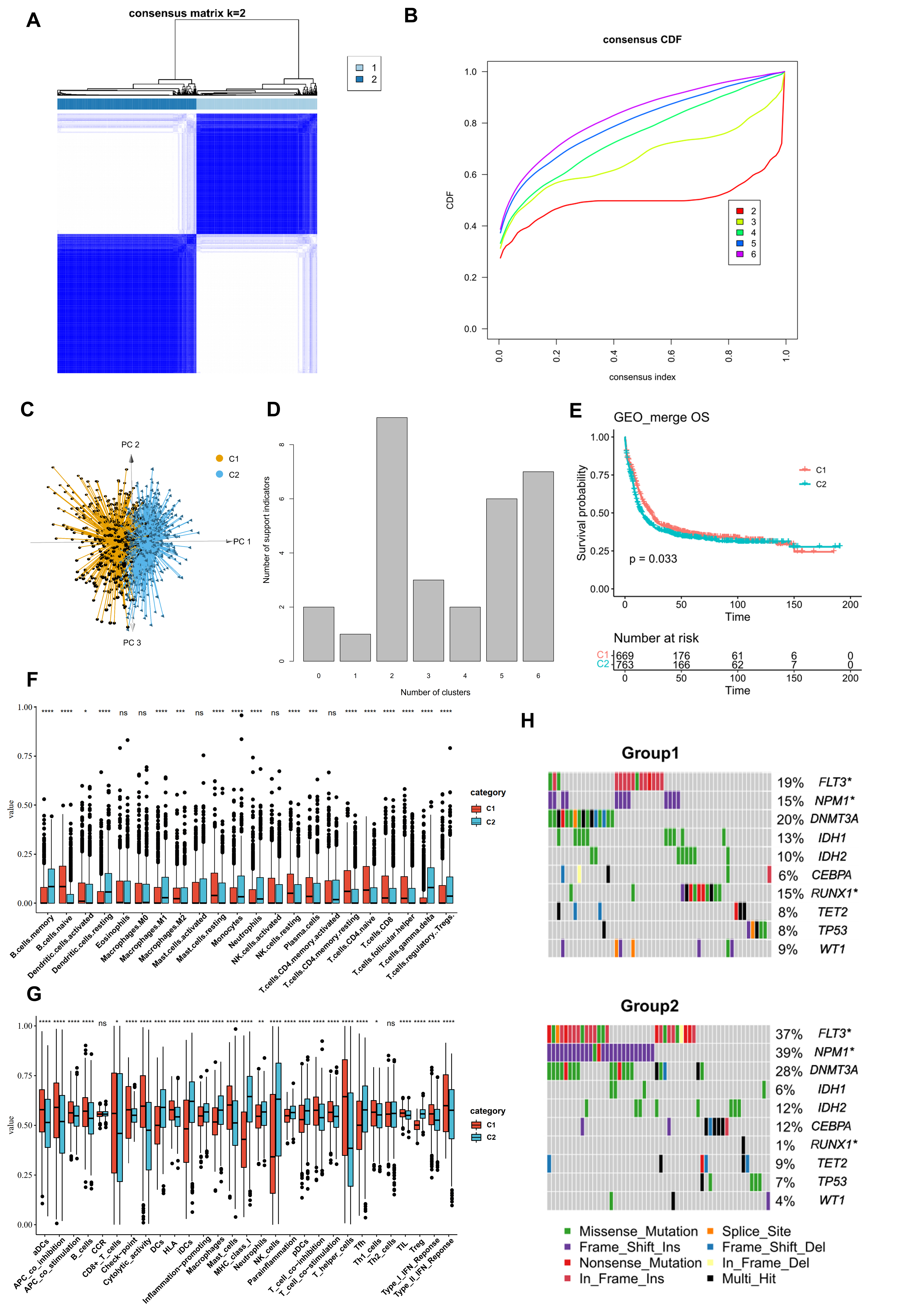

Supplement: Supplementary file 6 [file DataSheet1.zip › FigS2.TIF]

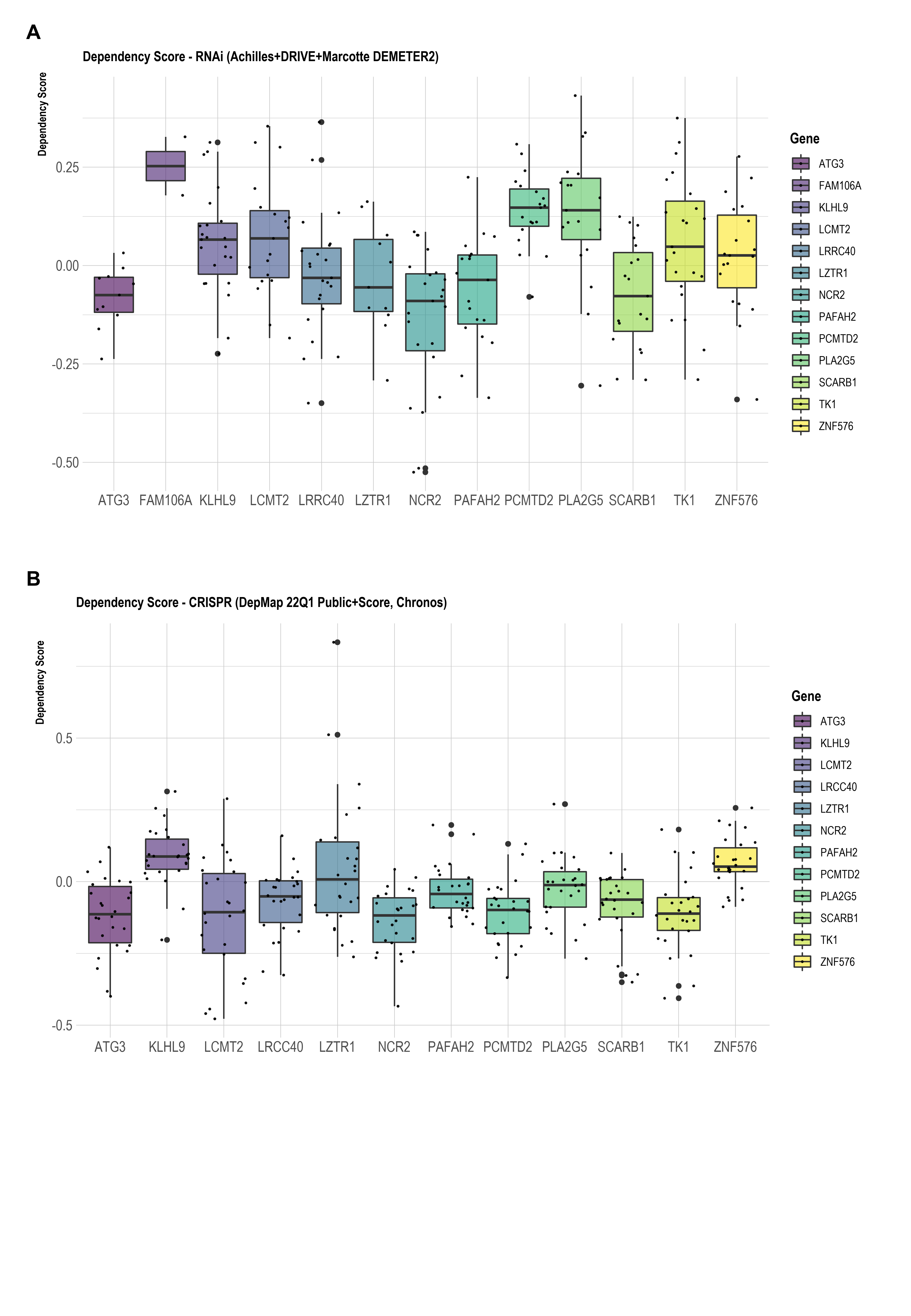

Supplement: Supplementary file 6 [file DataSheet1.zip › FigS5.TIF]

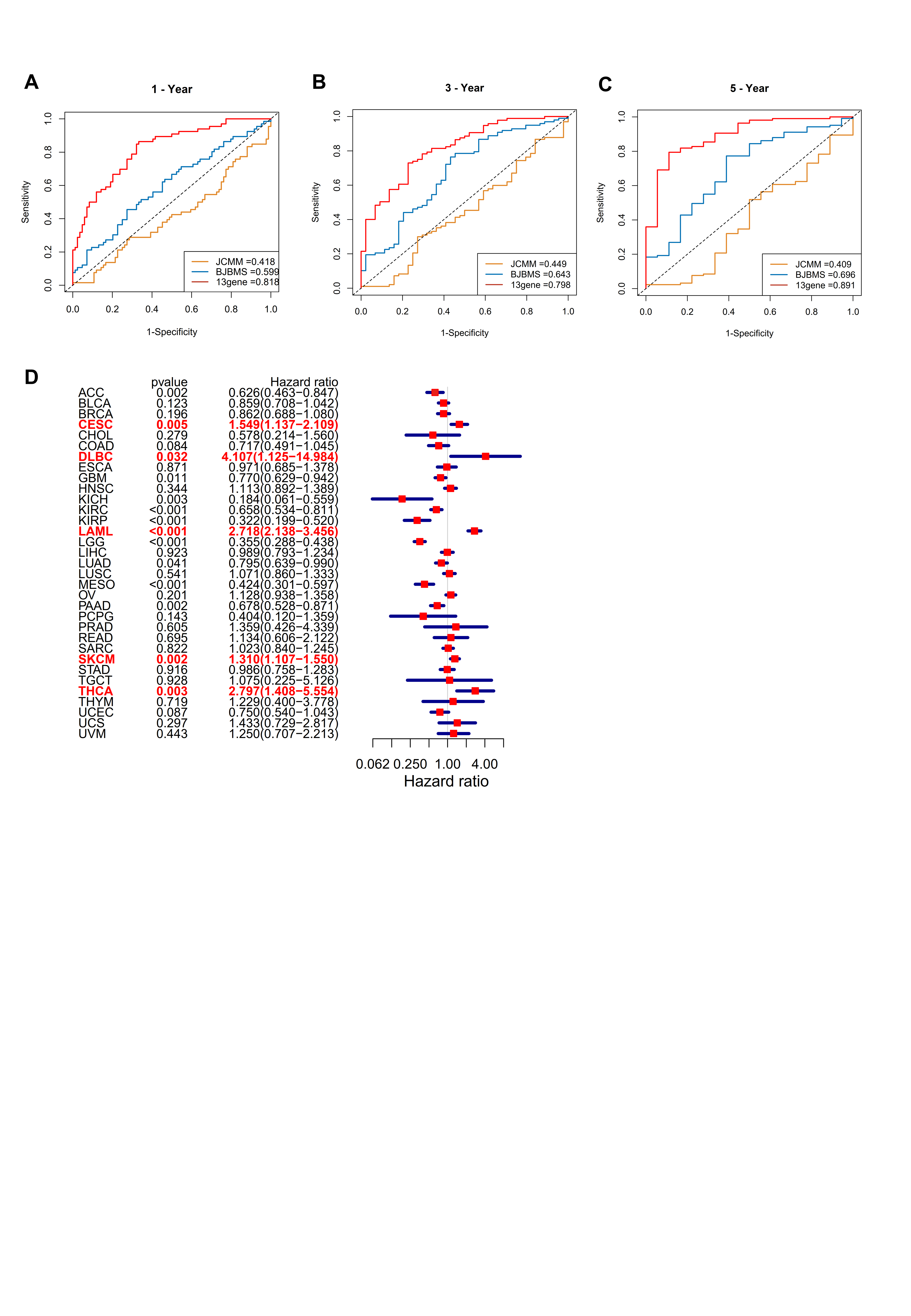

Supplement: Supplementary file 6 [file DataSheet1.zip › FigS7.TIF]
