# Supplementary material for: Comprehensive Analysis of a Ferroptosis Pattern and Associated Prognostic Signature in Acute Myeloid Leukemia
Source: Front Pharmacol. 2022 May 17;13:866325. doi: 10.3389/fphar.2022.866325 (PMC9152364; doi:10.3389/fphar.2022.866325)
Supplement: Supplementary file 6 [file DataSheet1.zip › Legends_for_Supplementary Figures and Tables.docx]

**Legends for Supplementary Figures and Tables:**

**Figure S1: Basic information of ferroptosis-related genes. (A)** Univariate cox result of 47 genes **(B)** PPI network of 47 genes **(C)** Waterfall map of TCGA mutations **(D)** 13 gene mutation status.

**Figure S2: Assessment of differences in prognosis, immune elements, and pathway enrichment between the two subtypes in TCGA cohort**. **(A)**: Correlation plot of 47 genes **(B)**: Consensus matrix of two subtypes **(C)**:CDF curve **(D)**: Indication barplot of k value used for consensus clustering. (**E)**: PCA plot of two subtypes. **(F)(G)**: Comparison of the difference in immune cell fraction and immune response between the two subtypes, with CibersortX and ssGSEA algorithm. （Wilcoxon tests） **(H)**: Waterfall maps of mutation status in two subtypes in TCGA.

**Figure S3: DEGs between TCGA two subtypes. (A)** Enrichment map of GSEA analysis in TCGA two subtypes. (**B)(C)** KEGG and GO analysis of DEGs between two subtypes in TCGA cohort. **(D)** Heatmap of top 10 genes with largest fold change in 193 genes.

**Figure S4: Survival Analysis of 13 genes in TCGA. (A-M)**: Survival analysis of 13 genes in TCGA(log-rank p<0.05),**(N)** Multivariate COX analysis concerning risk score, age, gender and top 10 mutations in TCGA.

**Figure S5: Dependency Score of 13 Genes in AML Cells from Depmap. (A):** Dependency score derived from RNA interference. **(B):** Dependency score derived from CRISPR knockdown.

**Figure S6: Construction of a Prognostic Nomogram in BeatAML datasets. (A**): A nomogram for clinical diagnosis based on age, mutation status and the risk score. **(B):** ROC curves for the nomogram. **(C-E)**: The calibration plots for predicting survival at 1, 2, and 3 years. The X-axis represents the predicted survival probability from the nomogram, and the y-axis represents the actual survival probability. **(F-H):** Decision curve analysis of the nomogram for 1-, 2- and 3-year risk. The x‐axis represents the threshold probability, and the y‐axis represents the net benefit. The green line represents the assumption that no patients died at 1, 2, or 3 years. The red line represents the assumption that all patients die at 1, 2, or 3 years and the blue line represents the prediction model of the nomogram.

**Figure S7: 13 Gene Score Compared with Newly Reported AML Prognostic Ferroptosis Risk Score and Validation in Pan-Cancer Cohorts.** **(A-C)**: Prognostic ROC curve AUC comparison in the 13-gene signatures with other latest ferroptosis risk signatures in training datasets (TCGA) for 1,3 and 5 years. **(D)**: Univariate COX analysis per cancer of a total of 10140 samples in 33 cancer type from TCGA project. Cancer type marked in red showed worse prognosis with higher score.

**Table1:** **Clincal Characteristics between Two Clusters Identified by Consensus Clustering.**

**Table2: Differential Gene between Two Clusters Identified by Consensus Clustering.**

**Table3: 13 Genes and Their Coefficients in This Model.**

**Table4: Clincal Characteristics between Two Risk Groups.**

**Table5: Primers of 13 genes of the model.**

**Table6: Ferroptosis-Related Genes Acquired from FerrDb for Further Analysis.**

**Table7: Univariate Cox Filtered Genes after DEG Filtering.**
